# Performance evaluation of clinical diagnostic kits for Group B *Streptococcus* available in China

**DOI:** 10.1128/spectrum.00137-26

**Published:** 2026-08-27

**Authors:** Rong Su, Xiaoli Chen, Shouneng Lu, Yujie Zhou, Yue Liu, Qiongzi Huang, Ying Zhang, Xinyan Zheng, Mengting Li, Wangheng Hou, Kunyu Yang, Huiming Ye, Yunfeng Lv, Zimin Tang, Shengxiang Ge

**Affiliations:** 1 State Key Laboratory of Vaccines for Infectious Diseases, Xiang An Biomedicine Laboratory, School of Public Health & School of Life Sciences, Xiamen University 554989, Xiamen, Fujian, China; 2 Department of Laboratory Medicine, Fujian Key Clinical Specialty of Laboratory Medicine, Women and Children’s Hospital, School of Medicine, Xiamen University 599486, Xiamen, China; 3 Center for Medical Device Evaluation, National Medical Products Administration 724304, Beijing, China; NHLS Tygerberg/Stellenbosch University, Cape Town, Western Cape, South Africa

**Keywords:** Group B *Streptococcus*, diagnostic kits, pregnancy, serotypes, sensitivity

## Abstract

Group B *Streptococcus* (GBS) is a significant pathogen capable of causing postpartum bacteremia in pregnant women and vertical transmission to neonates, which can result in fatal outcomes. Therefore, prenatal screening for vaginal GBS colonization is essential for preventing neonatal transmission. This study conducted an external quality assessment to evaluate the performance of 12 commercially available GBS detection kits in China. A total of 12 GBS detection kits (three antigen-based and nine nucleic acid-based) were evaluated using a reference panel comprising 11 GBS serotypes. The limit of detection (LOD) and repeatability were assessed for the antigen kits, and the LOD was determined for the nucleic acid detection kits. Nucleic acid testing achieved higher sensitivity than antigen-based methods. Antigen detection kits exhibited sensitivities ranging from 10⁴ to 10⁷ colony-forming units (CFU)/mL, with repeatability of at least 95%. Nucleic acid detection kits demonstrated sensitivities ranging from 10² to 10⁵ CFU/mL. Notably, certain kits showed reduced detection efficiency for serotypes Ia, III, VIII, and IV. The diagnostic performance of antigen detection kits and nucleic acid detection kits for detecting different serotypes varied. These findings underscore the potential for serotype-related analytical bias in clinical practice and emphasize the necessity of comprehensive kit validation.

**IMPORTANCE:**

Group B *Streptococcus* (GBS) is a leading cause of severe infections in newborns, often transmitted from colonized mothers during childbirth. Accurate prenatal screening is therefore critical. In China, numerous rapid test kits are used for GBS detection, but whether they perform equally well across different bacterial types (serotypes) was unknown. Our study systematically evaluated 12 commercially available kits and found that some fail to reliably detect certain high-risk serotypes, including one strongly linked to fatal newborn infections. These findings underscore the potential for serotype-related analytical bias in clinical practice and emphasize the necessity of comprehensive kit validation, thereby supporting more accurate screening to protect maternal and neonatal health.

## INTRODUCTION

Group B *Streptococcus* (GBS) is a Gram-positive, β-hemolytic bacterium that typically occurs in pairs or short chains. GBS is a major causative agent of genital tract infections in pregnant women, associated with conditions such as urinary tract infections, chorioamnionitis, postpartum endometritis, and maternal sepsis. It is also implicated in adverse obstetric outcomes including preterm birth and premature rupture of membranes. Neonatal GBS infection—often acquired vertically during delivery—can lead to sepsis, which is characterized by high incidence, mortality, and disability rates. As a result, GBS has emerged as a significant public health concern affecting maternal and neonatal health worldwide. Global estimates indicate an 18% colonization rate of GBS among pregnant women (1). In 2015, an estimated 33,000 cases of invasive GBS disease occurred in pregnant or postpartum women, alongside more than 57,000 cases of fetal infection or stillbirth attributed to ascending GBS infection (2). In China, the colonization rate is reported to be 11.3% (3). Without intrapartum prophylaxis, 50–60% of infants born to GBS-colonized mothers become colonized, and 1–2% develop invasive disease (4). Given the significant health impact of GBS and the crucial role of prenatal screening in guiding antibiotic prophylaxis, it is essential to ensure the accuracy and reliability of GBS diagnostic tools. However, it is unclear whether the various commercially available GBS detection kits in China perform equivalently across the range of clinically prevalent serotypes.

GBS is classified into 11 serotypes (Ia, Ib, Ic, and II–IX) based on variations in its capsular polysaccharide antigens. The global distribution of these serotypes exhibits significant geographical variation (1, 5, 6), which correlates with differences in virulence and disease manifestations (7, 8). In China, the predominant serotypes are Ia, Ib, II, III, and V. Notably, serotype III is strongly associated with invasive neonatal disease and elevated mortality rates (6).

The U.S. Centers for Disease Control and Prevention (CDC) guidelines recommend universal rectovaginal GBS screening for all pregnant women between 35 and 37 weeks of gestation. Individuals testing positive should receive intrapartum antibiotic prophylaxis to prevent vertical transmission (9). Since the implementation of this strategy in the USA, the incidence of early-onset GBS disease (EOGBS) decreased by 87% by 2015 compared with the 1990s (10). Chinese clinical guidelines also recommend universal GBS screening at 35–37 weeks of gestation. Additionally, pregnant women with GBS bacteriuria or who have had a previous infant with invasive GBS disease are considered colonized and should receive intrapartum antibiotic prophylaxis regardless of their screening results (11). Therefore, early prenatal screening and accurate diagnosis are critical for enabling timely intervention to protect maternal and neonatal health. Although microbial culture remains the gold standard for GBS detection, it requires 24–48 h of incubation to isolate monoclonal colonies, followed by confirmatory identification using mass spectrometry. Furthermore, competitive inhibition from vaginal and rectal commensal flora can lead to false-negative results (12). In contrast, quantitative real-time polymerase chain reaction (qPCR) enables detection of GBS within 1–2 h, significantly shortening the turnaround time for identifying both infected and colonized patients. Immunochromatographic assays offer another rapid alternative, providing results in 10–15 min without requiring specialized training. Their operational simplicity and rapid output make them particularly suitable in scenarios requiring immediate decisions regarding antibiotic prophylaxis.

Currently, 12 companies in the Chinese market offer rapid diagnostic kits for GBS, including three colloidal gold-based immunochromatographic antigen detection kits and nine fluorescence-based qPCR nucleic acid detection kits. While these kits enable rapid screening, GBS serotypes differ in key characteristics—such as virulence gene expression (13–18), capsular polysaccharide structure (19), and antigenic epitopes (20, 21)—which may affect the analytical sensitivity or specificity of target-dependent assays. This variability can lead to missed detections and, consequently, to misdiagnosis or inappropriate clinical management. Consequently, ensuring high accuracy, sensitivity, and broad-spectrum coverage of commercially available diagnostic kits is critical. Most published evaluations of GBS detection methods have focused on two main aspects: comparing the sensitivity and specificity of different methodologies (e.g. culture, PCR, and antigen testing) (22) and comparing the performance of various commercial diagnostic platforms using clinical specimens (23–25). However, none of these studies have systematically investigated the impact of serotype diversity on detection performance. Secondly, while external quality assessments (EQA) have been conducted on certain commercial GBS kits (26), these evaluations have generally employed a limited number of strains and have not covered all the kits available on the Chinese market. Thirdly and most critically, no study has examined whether the detection efficiency of kits marketed in China varies for serotypes that are highly clinically relevant in China, such as serotype III. These research gaps raise concerns that some widely used kits may harbor unrecognized serotype-related analytical biases, which could lead to missed diagnoses in high-risk populations.

Therefore, a systematic, manufacturer-independent evaluation using reference materials encompassing multiple serotypes is urgently needed to objectively assess the reliability of these kits across a broad spectrum. In this study, we employed laboratory-developed GBS reference materials representing multiple serotypes, alongside culture-based methods (the gold standard) to evaluate the performance of the major GBS detection kits currently available in China.

## MATERIALS AND METHODS

### Preparation and validation of reference materials

**Fig 1:**
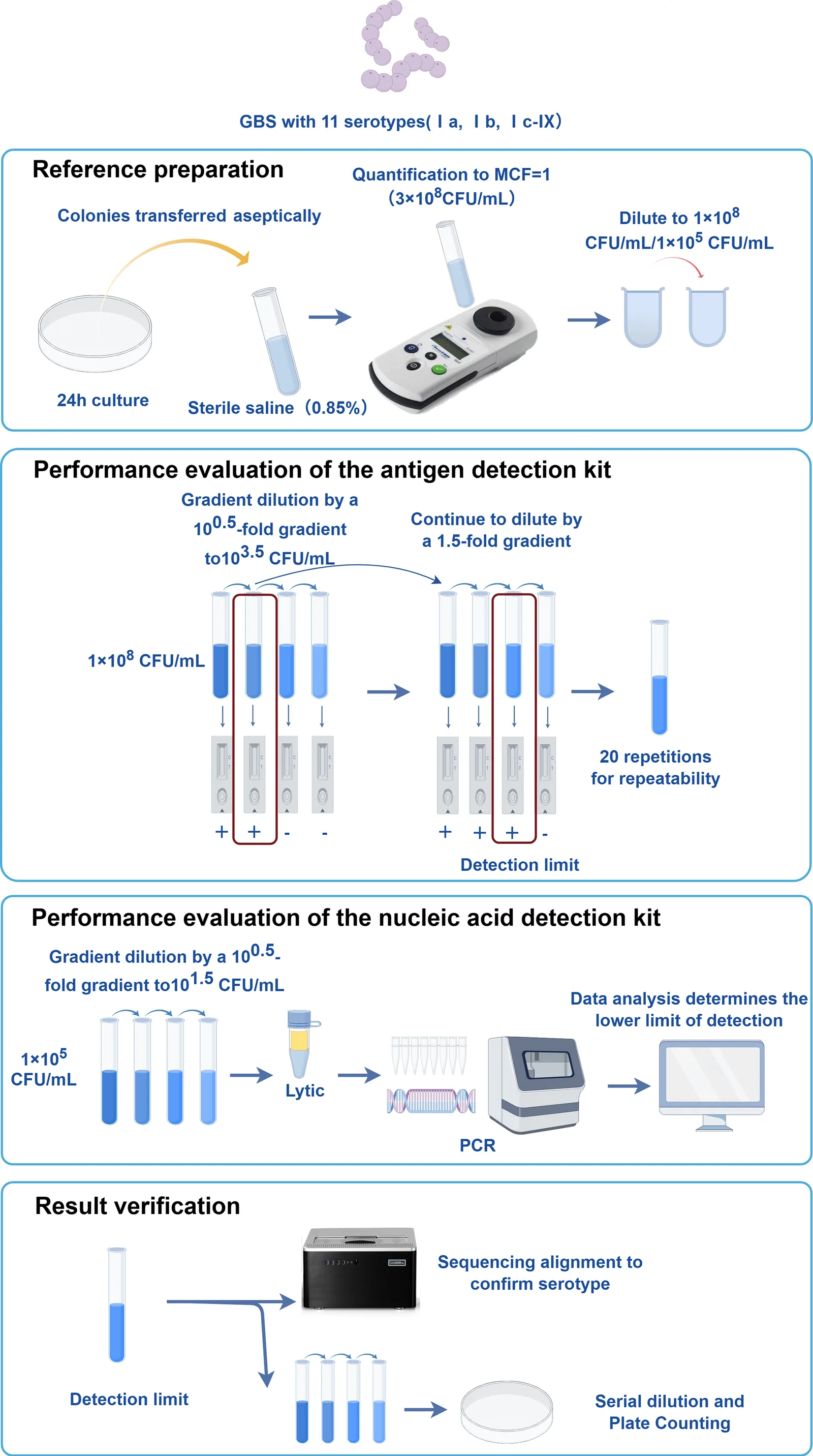
"Flowchart for the preparation of reference samples and performance evaluation of the kit.

The reference materials utilized in this study were developed and provided by the project team. These materials comprised 11 serotypes (Ia, Ib, Ic, and II–IX) of inactivated GBS at standardized concentrations (CFU/mL) (Table S1; Fig. 1), with *Escherichia coli* serving as a negative control. After quantification by the plate counting method, the bacterial suspensions were inactivated with β-propiolactone (BPL) at a ratio of 1:2,000 (vol/vol) overnight at 4°C, and the reaction was terminated by incubation at 37°C (27). To confirm complete inactivation, the BPL-treated bacterial suspensions were inoculated onto Columbia blood agar plates and incubated aerobically at 37°C for 48 h. Batches were considered fully inactivated and eligible for use only when no bacterial growth was observed on the plates. The homogeneity and equivalence of GBS concentrations across serotypes were verified through qPCR targeting the housekeeping gene adhP (28). Additionally, Oxford Nanopore whole-genome sequencing was employed to confirm the serotype identity and genetic integrity of all reference strains.

### Group B *Streptococcus* clinical diagnostic kits

The analytical performance evaluation included a total of twelve commercial kits: three antigen detection kits and nine nucleic acid detection kits (Table 1; Table S2), all of which are qualitative detection kits. Most kits incorporate proprietary nucleic acid extraction reagents and employ thermal lysis for crude nucleic acid extraction. In contrast, one kit employs an alkaline lysis method without a heating step, while the other two kits require the separate purchase of company-specific, magnetic bead–based extraction products. Serially diluted reference samples were prepared, and all kits were operated in strict accordance with manufacturer instructions to evaluate the LOD and reproducibility of antigen detection kits (Fig. 1). Results were interpreted according to the cycle threshold (Ct) values or amplification curve thresholds provided in each kit’s instructions. For immunochromatographic kits, results were read by visual inspection, with the appearance of a test line (T-line) being considered positive.

**TABLE 1:** Basic information about the test kit

Kit	Method	Company	Target	Lysis/extraction method
1	Immune colloidal gold technique	HuaAo	Unknown	Chemical lysis
2		Genfocus	Unknown	Chemical lysis
3		Weimi	Unknown	Chemical lysis
4	Real-time polymerase chain reaction	TIB	CAMP	Heat lysis
5		BioPerfectus	CAMP	Heat lysis
6		Biotron	CAMP	Magnetic bead extraction
7		TianLong	CAMP	Heat lysis
8		COYOTE	Unknown	Alkaline cleavage (no heating required)
9		DaAn Gene	Unknown	Magnetic bead extraction
10		BioChain	CAMP	Heat lysis
11		Hybribio	Unknown	Heat lysis
12		Liferiver	Unknown	Heat lysis

### Evaluation criteria and analysis of results

This study employed a single-blind design. All evaluations were performed independently by multiple laboratory personnel, and results were averaged. The LOD was defined as the lowest concentration at which the target was consistently detected in serially diluted reference samples. Consistent non-detection was required at the next lower dilution. Repeatability was assessed by testing the LOD concentration across 20 independent replicates.

### Statistical analysis

Data were analyzed using SPSS Statistics (version 25.0), and figures were generated using GraphPad Prism (version 9.5). As the LOD values were not normally distributed, variability in kit performance across serotypes was evaluated using the interquartile range (IQR). Pairwise *t*-tests were applied to assess consistency between different reference samples.

## RESULTS

### Reference material validation

A reference panel comprising 11 GBS serotypes was successfully established. All strains were confirmed as GBS by microbial mass spectrometry, with *Escherichia coli* used as a negative control. qPCR analysis of the housekeeping gene adhP across different serotypes at an equivalent concentration (3 × 10⁸ CFU/mL) revealed no statistically significant differences in Ct values (*P* > 0.05) (Fig. S1), confirming consistent detection efficiency. The accuracy of reference material concentrations and serotype identities was further validated. Oxford Nanopore whole-genome sequencing confirmed the taxonomic assignment and serotype of each strain. Additionally, plate counting verified that the bacterial concentrations of all samples were within the expected order of magnitude.

### Antigen detection kits

**Fig 2:**
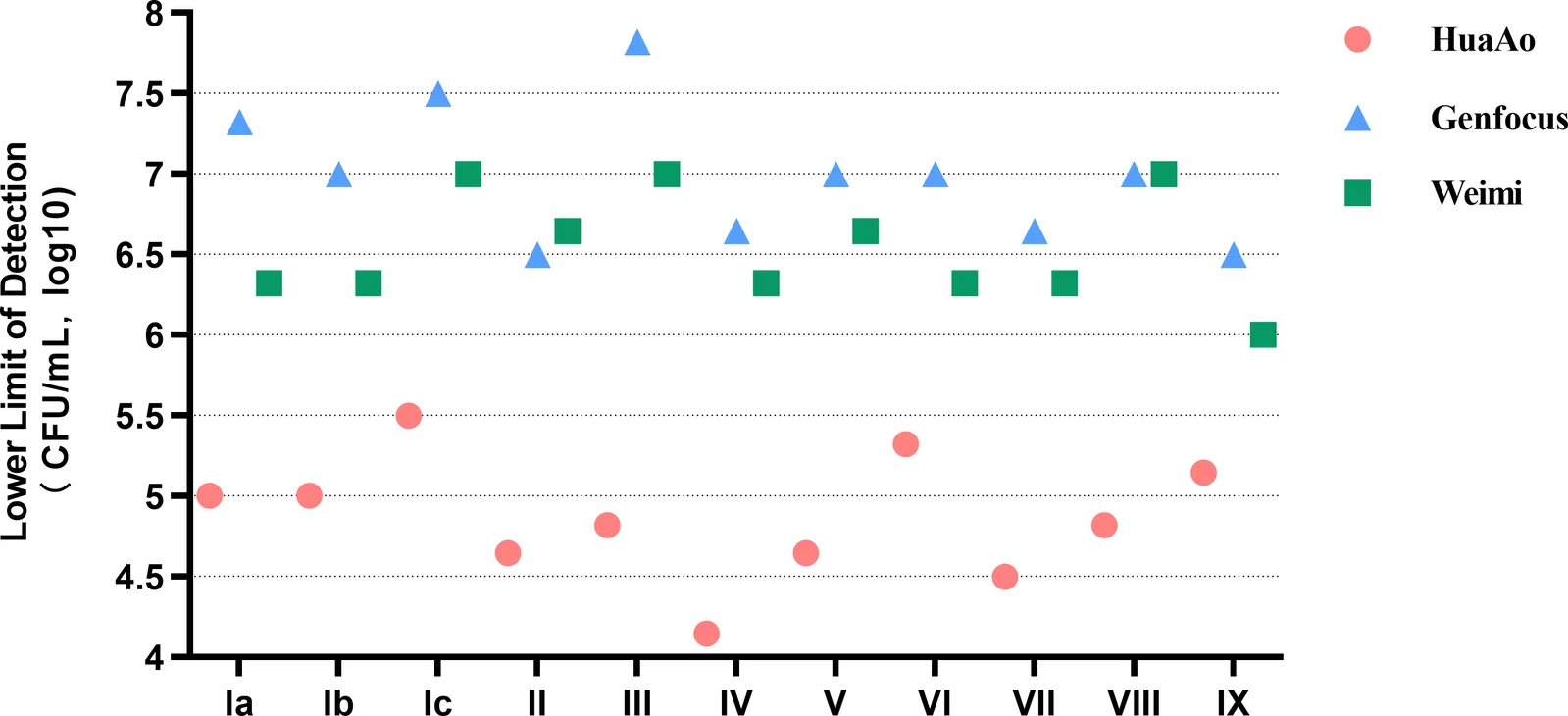
Detection limits of antigen kits across serotypes.

Commercial antigen detection kits were used to analyze GBS reference strains and *E. coli* controls. All three antigen detection kits successfully identified all GBS serotype strains as positive while correctly yielding negative results for samples containing *E. coli*. The LOD was evaluated using serially diluted reference standards (Table S3; Fig. 2). The kits exhibited a range of sensitivities. One kit (Kit 1) demonstrated the highest sensitivity with an LOD of 10⁴–10⁵ CFU/mL and 100% reproducibility. The other two kits (Kits 2 and 3) showed higher LODs of 10⁶–10⁷ CFU/mL, with reproducibility exceeding 95% for both. Serotype-specific variations in detection efficiency were observed. Kit 1 performed best for serotype IV but showed reduced efficiency for serotype Ic. Kit 2 was most effective for serotypes II and IX, but less sensitive for serotype III. Kit 3 demonstrated optimal detection of serotype IX, with no notably weak performance against any serotype. The narrow IQR of the LOD values across all three kits (Table S3) indicates stable and consistent analytical performance.

### Nucleic acid detection kits

**Fig 3:**
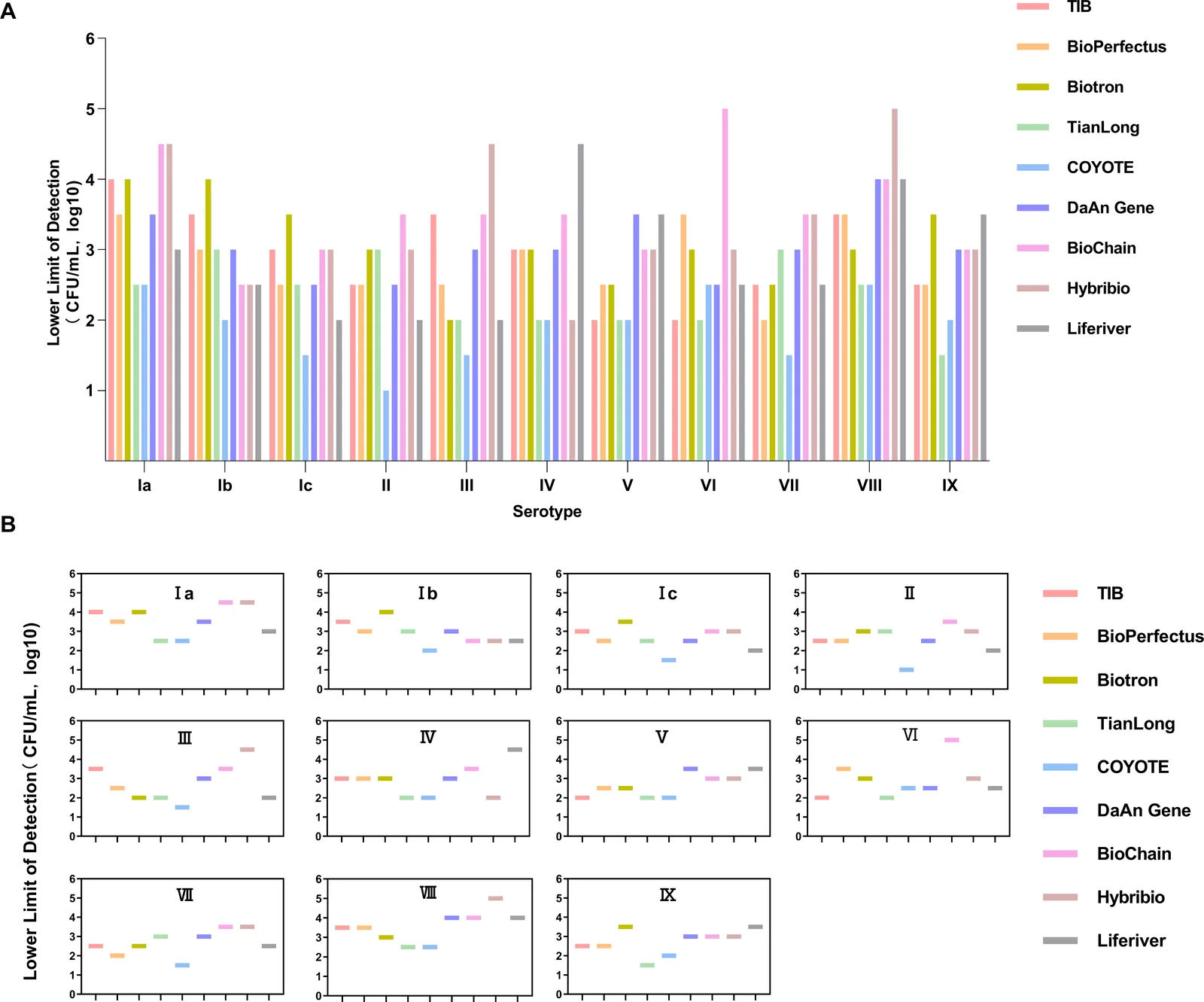
Nucleic acid detection kits. (**A**) Detection limits of nucleic acid kits stratified by serotype. (**B**) Detection limits of 11 serotypes across kits.

All nine commercial nucleic acid detection kits correctly identified all GBS serotype strains as positive and yielded negative results for samples containing *E. coli*. Evaluation using serially diluted reference standards demonstrated that the LOD for the nine kits ranged from 10² to 10⁵ CFU/mL (Table S4; Fig. 3). While all kits successfully detected high-concentration samples (10⁵ CFU/mL), detection performance was inconsistent for low-concentration samples. Manufacturer-reported sensitivity was ≤10⁴ CFU/mL for all kits. Among the 99 tests performed (11 serotypes × 9 kits), 93.93% (93/99) met this claimed sensitivity threshold.

Analysis of the kits’ LOD, as specified by the manufacturers (Fig. 3A), revealed notable performance variations. Kits 4–9 demonstrated higher sensitivity, with Kit 8 exhibiting the highest overall sensitivity (LOD for all serotypes < 10².⁵ CFU/mL). In contrast, three kits showed comparatively lower sensitivity: Kit 11 displayed LOD values > 10⁴ CFU/mL for 27.3% of serotypes (3/11: Ia, III, VIII); Kit 10 showed LOD > 10⁴ CFU/mL for 18.2% of serotypes (2/11: Ia, VI); and Kit 12 exhibited LOD > 10⁴ CFU/mL for 9.1% of serotypes (1/11: IV). Performance variability was most pronounced for these three kits when testing different serotypes. The serotype-dependent variations in detection sensitivity observed for these kits could impact clinical detection rates, especially for serotypes that demonstrated higher LODs in this study.

Analysis of serotype-specific detection limits across the nine kits (Fig. 3B) revealed that six serotypes (Ib, Ic, II, V, VII, and IX; 54.5%) were consistently detected at concentrations ≤10⁴ CFU/mL by all kits. In contrast, detection sensitivity for other serotypes was variable and kit-dependent: two kits failed to detect serotype Ia at 10⁴ CFU/mL, whereas one kit each failed to detect serotypes III, IV, VI, and VIII at this concentration. This variability may lead to missed detections, particularly in regions where these serotypes are prevalent. Notably, detection capabilities for serotypes Ia and III exhibited the greatest heterogeneity among kits, indicating elevated risk of diagnostic escape for these clinically relevant serotypes.

## DISCUSSION

GBS is an opportunistic pathogen, with pregnant women and neonates representing high-risk populations for invasive infection. Vaginal-rectal colonization in pregnant women can lead to postpartum infections, bacteremia, endocarditis, and skin/soft tissue infections. Vertical transmission during delivery may cause neonatal sepsis, meningitis, and pneumonia—conditions associated with a pooled case fatality risk of 8.4% (29). Consequently, numerous national guidelines underscore the necessity of prenatal GBS screening (9, 11, 30–33).

To support clinical diagnosis and infection control, multiple Chinese manufacturers have developed GBS detection kits utilizing antigen-based and nucleic acid-based methods. However, standardized reference materials suitable for cross-methodological evaluation are lacking. In this study, β-propiolactone (BPL)-inactivated whole bacterial cultures were employed as multivalent serotype reference materials. BPL inactivates bacteria effectively while largely preserving cell membrane integrity and the activity of intracellular components (27, 34), thereby better simulating the matrix of clinical specimens. These reference materials retain intact bacterial genomes, covering all potential detection targets, which ensures impartial and broadly applicable performance assessments. They are therefore suitable for the parallel evaluation of both antigen- and nucleic acid-based detection methods.

As noted earlier, most published evaluations of GBS detection methods have focused on comparing the overall sensitivity and specificity of different methodologies (e.g., culture vs. PCR) or commercial platforms using clinical samples. However, the influence of serotype diversity on detection performance has been systematically investigated only to a limited extent. For example, Honest (35) analyzed 29 studies involving 15,691 pregnant women and concluded that real-time PCR is the most accurate rapid detection method for GBS. Nielsen (23) directly compared two commercial PCR kits (Xpert GBS and GenomEra GBS), revealing differences in sensitivity when tested on clinical specimens. In China, Chen (26) conducted a small-scale EQA for GBS molecular diagnostics using only serotype II. Their results indicated that although all 43 participating laboratories successfully detected high-concentration samples (20,000 copies/mL, ≈20,000 CFU/mL) using various commercial PCR kits—a finding consistent with our observations—only 15 correctly identified low-concentration samples (≤200 copies/mL). Detection performance for low-concentration targets varied substantially among kits. For example, whereas kit 11 was reported in that study to exhibit relatively high sensitivity, our data showed a higher LOD (10³ CFU/mL) for serotype II. These collective findings underscore two persistent challenges in GBS diagnostics: reliable detection of low bacterial loads and considerable variability both among kits and across laboratories. Importantly, neither study explored whether such performance discrepancies could be linked to differences in GBS serotypes. These observations underscore the common challenges in detecting low bacterial loads and the performance variability across different kits. Evidence suggests that GBS serotypes vary in key characteristics such as virulence gene expression (13–18), capsular polysaccharide structure (19), and antigenic epitopes (20, 21). These biological differences likely contribute significantly to the observed variability in kit performance. Consequently, validating kit performance using only a single serotype may not accurately reflect its diagnostic capability across the spectrum of clinically relevant strains, potentially masking inherent detection biases.

The results of this study directly substantiate these performance discrepancies. Using a multi-serotype reference panel, systematic evaluation revealed notable variations in the detection limits among nucleic acid kits for different GBS serotypes. Marked differences in sensitivity were observed for serotypes Ia, III, IV, VI, and VIII across products (Fig. 3; Table S4). This finding has significant clinical implications. Serotypes I–V account for approximately 98% of global maternal colonization (36). Notably, serotype III constitutes 46.6% of invasive neonatal GBS disease in China and is associated with higher mortality (37). Therefore, the suboptimal detection of serotype III by certain kits, as observed in this study, may lead to missed identification of high-risk pregnancies in clinical practice. This could delay intrapartum antibiotic prophylaxis and increase the risk of early-onset GBS sepsis. Consequently, we emphasize the necessity of incorporating multi-serotype samples—particularly those reflecting local epidemiology and high-risk serotypes—into kit evaluation and optimization protocols.

All evaluated kits demonstrated accurate detection of high-concentration GBS samples across all serotypes, with performance comparable to that of internationally established assays such as GenePOC GBS LB and Xpert GBS (38, 39). However, performance was notably reduced for low-concentration samples (≤10³ CFU/mL). Among the nine nucleic acid detection kits, 70.7% (70/99) of serotype-specific measurements yielded a LOD below 10³ CFU/mL . For certain serotypes (e.g., Ia, III, IV, and VIII), however, the LODs for some kits exceeded 10⁴ CFU/mL (Table S4). This variability may be attributed to several factors. First, nucleic acid extraction efficiency differed among kits. For example, one kit employed chemical lysis, two kits required separate magnetic bead–based nucleic acid extraction reagents, and the remainder utilized proprietary thermal lysis–based methods for crude nucleic acid extraction. These methodological differences likely affect the efficiency of bacterial cell wall disruption, nucleic acid recovery, and purity (40), thereby influencing the detection of low-concentration targets. Second, the choice and conservation of detection targets may impact serotype-specific sensitivity. Only five kits in this study explicitly targeted the cfb gene (encoding CAMP factor); the targets for the remaining kits were not disclosed (Table 1). Studies have indicated that partial deletions can occur within the chromosomal region harboring the cfb gene in GBS (41, 42). Such structural variations may alter local DNA architecture or affect the length and integrity of extracted fragments, potentially interfering with the efficient amplification of limited template in low-concentration samples. This possibility is supported by the findings of Lai (43), who detected cfb deletions in all 19 clinical GBS isolates (all serotype III) examined. Together, these observations suggest that target sequence conservation or genomic context may be serotype-associated. In summary, serotype-specific variability, combined with the overall sensitivity and inhibitor tolerance of kits at low bacterial loads, represents a key determinant of clinical detection rates. Manufacturers should prioritize these factors during product optimization. Furthermore, molecular assays relying solely on the cfb gene may yield false-negative results. We therefore recommend that manufacturers adopt multi-target designs (e.g., simultaneous detection of cfb and scpB) and standardize extraction protocols to improve the detection of low-concentration samples and high-risk serotypes. Wider implementation of automated extraction and detection systems would also help minimize operator-dependent variability and improve inter-laboratory consistency.

During the validation of nucleic acid detection kits, we observed that samples with Ct values exceeding the manufacturer’s specified cutoff threshold frequently exhibited few but detectable colonies on blood agar, indicating culture positivity. This suggests that samples yielding Ct values near or above the manufacturer’s recommended threshold should be confirmed using conventional culture methods to avoid false-negative interpretations. Furthermore, the American Society for Microbiology (ASM) guidelines recommend pre-enrichment of samples prior to PCR testing to improve detection sensitivity (44). In clinical practice, pre-enrichment of vaginal-rectal swabs may increase bacterial load and significantly enhance GBS detection rates. Similarly, given the inherently lower sensitivity of immunochromatographic assays, sample pre-enrichment is also advised before using antigen-based kits to improve diagnostic accuracy.

All diagnostic kits evaluated in this study are qualitative in nature. While qualitative testing typically fulfills the requirement for routine antenatal screening, quantitative or semi-quantitative data could provide critical information for specific clinical decisions. For example, a meta-analysis demonstrated that GBS bacteriuria in pregnancy—particularly with colony counts >10⁴ CFU/mL—correlates with a significantly increased risk of preterm delivery (45). Accordingly, clinical guidelines from both the USPSTF (46) and Chinese experts (11) recommend intrapartum antibiotic prophylaxis when GBS colony counts in urine exceed this threshold. Although the present study did not assess the quantitative capabilities of the kits, future developments that enable semi-quantitative reporting (e.g., bacterial load estimation via Ct values) could offer clinicians a more precise tool for patient risk stratification and support personalized management decisions.

This study has several limitations. First, the performance evaluation was conducted using bacterial suspension samples under controlled laboratory conditions. These laboratory conditions may not fully replicate the complexity of clinical specimens, which often contain inhibitors such as vaginal mucus, blood, or other contaminants that could affect assay accuracy. Second, the long-term stability of the kits—including batch-to-batch consistency and reagent durability—was not assessed. Future multicenter clinical trials utilizing prospectively collected vaginal-rectal swabs are warranted to further validate these findings under real-world conditions.

### Conclusions

This study evaluated the analytical performance of three commercially available GBS antigen detection kits and nine nucleic acid detection kits in China using standardized reference materials under laboratory conditions. A detailed analysis of detection limits was performed, with the results stratified by both kit manufacturer and GBS serotype. The detection sensitivity of antigen-based kits was consistently lower than that of nucleic acid-based tests, reflecting inherent limitations of immunological methodologies. While most nucleic acid detection kits demonstrated high analytical sensitivity, certain products exhibited suboptimal performance, particularly for specific serotypes, indicating a need for further optimization. To our knowledge, this is the first systematic performance evaluation in China to demonstrate that certain diagnostic kits exhibit markedly reduced sensitivity toward specific high-risk GBS serotypes, such as Ia and III. These findings offer valuable evidence to support manufacturers in optimizing their products and assist clinical laboratories in selecting appropriate tests based on local serotype epidemiology.

However, as this evaluation was conducted within a single laboratory setting, the generalizability of the results may be limited. To ensure diagnostic reliability and patient safety, we recommend strengthening quality control measures and regulatory supervision of GBS nucleic acid detection kits. This should include regular randomized testing of commercially available kits to verify claimed sensitivity and accuracy, along with implementing corrective actions for products that fail to meet established quality standards. Ultimately, such measures will contribute to more reliable prenatal GBS screening, thereby reducing the burden of neonatal invasive disease in China.

## Supplementary Material

Reviewer comments

## SUPPLEMENTAL MATERIAL

Tables S1 to S4; Fig. S1 spectrum.00137-26-s0001.docx Kits test results.

OPEN PEER REVIEW reviewer-comments.pdf An accounting of the reviewer comments and feedback.

ASM does not own the copyrights to Supplemental Material that may be linked to, or accessed through, an article. The authors have granted ASM a non-exclusive, world-wide license to publish the Supplemental Material files. Please contact the corresponding author directly for reuse.
