# Supplementary material for: Performance evaluation of clinical diagnostic kits for Group B Streptococcus available in China
Source: Microbiol Spectr. 2026 Aug 27;14(10):e00137-26. doi: 10.1128/spectrum.00137-26 (PMC13689486; doi:10.1128/spectrum.00137-26)
Supplement: Supplementary file 1 [file reviewer-comments.pdf]

AMERICAN SOCIETY FOR MICROBIOLOGY | Microbiology Spectrum

Performance evaluation of clinical diagnostic kits for Group B streptococcus available in China

Rong Su, Xiao-Li Chen, Shou-neng Lu, Yu-jie Zhou, Yue Liu, Qiong-zi Huang, Ying Zhang, Xin-yan Zheng, Meng-ting Li, Wang-Heng Hou, Kun-yu Yang, Hui-Ming Ye, Yun-feng Lv, Zi-Min Tang, and Shengxiang Ge

Corresponding Author(s): Zi-Min Tang, Xiamen University

Review Timeline:

Submission Date:	January 13, 2026
Editorial Decision:	January 24, 2026
Revision Received:	January 28, 2026
Editorial Decision:	June 16, 2026
Revision Received:	June 20, 2026
Accepted:	June 23, 2026

Editor: Kessendri Reddy

Reviewer(s): The reviewers have opted to remain anonymous.

Transaction Report:

(Note: With the exception of the correction of typographical or spelling errors that could be a source of ambiguity, letters and reports are not edited. The original formatting of letters and referee reports may not be reflected in this compilation.)

DOI: https://doi.org/10.1128/spectrum.00137-26

Re: Spectrum00137-26 (Performance evaluation of clinical diagnostic kits for Group B streptococcus available in China)

Dear Mx. Zi-Min Tang:

Thank you for the privilege of reviewing your work. Below you will find my comments, instructions from the Spectrum editorial office, and the reviewer comments.

This kind of analysis, comparing different commercially available kits, is important. However, the technical quality of this paper (one of the main focuses of Microbiology Spectrum) is lacking. Although several kits were employed, further details of these kits including the methods they require, need to be provided as readers will not necessarily be familiar with these various assays. Suggest that this information either be included succinctly in the Methods section of this manuscript or as supplementary material, before the article can be sent out for review.

Please return the manuscript within 60 days; if you cannot complete the modification within this time period, please contact me. If you do not wish to modify the manuscript and prefer to submit it to another journal, notify me immediately so that the manuscript may be formally withdrawn from consideration by Spectrum.

Revision Guidelines
To submit your modified manuscript, log into the submission site at https://spectrum.msubmit.net/cgi-bin/main.plex. Go to Author Tasks and click the appropriate manuscript title to begin. The information you entered when you first submitted the paper will be displayed; update this as necessary. Note the following requirements:

• Upload point-by-point responses to the issues raised by the reviewers in a file named "Response to Reviewers," NOT in your cover letter.
• Upload a compare copy of the manuscript (without figures) as a "Marked-Up Manuscript" file.
• Upload a clean .DOC/.DOCX version of the revised manuscript and remove the previous version.
• Each figure must be uploaded as a separate, editable, high-resolution file (TIFF or EPS preferred), and any multipanel figures must be assembled into one file.
• Any supplemental material intended for posting by ASM should be uploaded with their legends separate from the main manuscript. You can combine all supplemental material into one file (preferred) or split it into a maximum of 10 files with all associated legends included.

For complete guidelines on revision requirements, see our Submission and Review Process webpage. Submission of a paper that does not conform to guidelines may delay acceptance of your manuscript.

Data availability: ASM policy requires that data be available to the public upon online posting of the article, so please verify all links to sequence records, if present, and make sure that each number retrieves the full record of the data. If a new accession number is not linked or a link is broken, provide Spectrum production staff with the correct URL for the record. If the accession numbers for new data are not publicly accessible before the expected online posting of the article, publication may be delayed; please contact production staff (Spectrum@asmusa.org) immediately with the expected release date.

Publication Fees: For information on publication fees and which article types are subject to charges, visit ourwebsite. If your manuscript is accepted for publication and any fees apply, you will be contacted separately about payment during the production process; please follow the instructions in that e-mail. Arrangements for payment must be made before your article is published.

ASM Membership: Corresponding authors may join or renew ASM membership to obtain discounts on publication fees. Need to upgrade your membership level? Please contact Customer Service at Service@asmusa.org.

The ASM Journals program strives for constant improvement in our submission and publication process. Please tell us how we can improve your experience by taking this quick Author Survey.

Thank you for submitting your paper to Spectrum.

Sincerely,
Kessendri Reddy
Editor
Microbiology Spectrum

Re: Spectrum00137-26R1 (Performance evaluation of clinical diagnostic kits for Group B streptococcus available in China)

Dear Mx. Zi-Min Tang:

Thank you for the privilege of reviewing your work. Below you will find my comments, instructions from the Spectrum editorial office, and the reviewer comments.

Thank you for providing the additional information. Minor comments below:
- in Table S3, please report the lower limit of detection in more understandable terms, e.g. replace 1.00E+07 with 10 000 000 or 10 to the power of 7
- Lines 65-67 seem to be incorrect, can you double check on this fact and explain it more comprehensively if the fact is correct (33000 maternal infections per year resulting in 57000 adverse pregnancy outcomes)?
- Lines 80-83 state that Chinese guidelines recommend universal screening, but the specific indications given are not consistent with a universal screening approach. Please clarify. In addition, screening cannot be done too early in the pregnancy as colonisation can fluctuate during the pregnancy.
- Line 205 indicates a mortality rate of 8.4% to be high. Surely this is an underestimate? Please review this figure and correct or explain as appropriate.

Please return the manuscript within 60 days; if you cannot complete the modification within this time period, please contact me. If you do not wish to modify the manuscript and prefer to submit it to another journal, notify me immediately so that the manuscript may be formally withdrawn from consideration by Spectrum.

Revision Guidelines
To submit your modified manuscript, log into the submission site at https://spectrum.msubmit.net/cgi-bin/main.plex. Go to Author Tasks and click the appropriate manuscript title to begin. The information you entered when you first submitted the paper will be displayed; update this as necessary. Note the following requirements:

• Upload point-by-point responses to the issues raised by the reviewers in a file named "Response to Reviewers," NOT in your cover letter.
• Upload a compare copy of the manuscript (without figures) as a "Marked-Up Manuscript" file.
• Upload a clean .DOC/.DOCX version of the revised manuscript and remove the previous version.
• Each figure must be uploaded as a separate, editable, high-resolution file (TIFF or EPS preferred), and any multipanel figures must be assembled into one file.
• Any supplemental material intended for posting by ASM should be uploaded with their legends separate from the main manuscript. You can combine all supplemental material into one file (preferred) or split it into a maximum of 10 files with all associated legends included.

For complete guidelines on revision requirements, see our Submission and Review Process webpage. Submission of a paper that does not conform to guidelines may delay acceptance of your manuscript.

Data availability: ASM policy requires that data be available to the public upon online posting of the article, so please verify all links to sequence records, if present, and make sure that each number retrieves the full record of the data. If a new accession number is not linked or a link is broken, provide Spectrum production staff with the correct URL for the record. If the accession numbers for new data are not publicly accessible before the expected online posting of the article, publication may be delayed; please contact production staff (Spectrum@asmusa.org) immediately with the expected release date.

Publication Fees: For information on publication fees and which article types are subject to charges, visit our website. If your manuscript is accepted for publication and any fees apply, you will be contacted separately about payment during the production process; please follow the instructions in that e-mail. Arrangements for payment must be made before your article is published.

ASM Membership: Corresponding authors may join or renew ASM membership to obtain discounts on publication fees. Need to upgrade your membership level? Please contact Customer Service at Service@asmusa.org.

The ASM Journals program strives for constant improvement in our submission and publication process. Please tell us how we can improve your experience by taking this quick Author Survey.

Thank you for submitting your paper to Spectrum.

Sincerely,
Kessendri Reddy
Editor

Reviewer #1 (Comments for the Author):

Thank you for the opportunity to review this manuscript. The authors have presented a well-structured and significant piece of work. I offer the following suggestions to further strengthen the manuscript for publication.
Introduction:
To enhance clarity for the reader, I recommend explicitly stating the research question and hypothesis early in the introduction.
Situating the study within the existing body of knowledge is crucial. I suggest incorporating a brief review of previous relevant works to provide context for the current research.
The manuscript would benefit from a more detailed explanation of the challenges and gaps in the current research that this study aims to address.
Methods:
Could you please provide more specific details regarding the methodology used?
I noticed a few minor grammatical errors throughout the manuscript. A thorough proofread would help to improve the overall readability.
I believe that addressing these points will significantly improve the manuscript's impact and clarity.

Response to Reviewer Comments on the manuscript Spectrum00137-26R1

We thank the reviewers for the positive comments. We have addressed all the reviewer's comments and revised the manuscript accordingly. To facilitate the navigation of this document, we have copied the reviewer's comments verbatim in **blue** and typed out our responses in **black**.

Editor

1. In Table S3, please report the lower limit of detection in more understandable terms, e.g. replace 1.00E+07 with 10 000 000 or 10 to the power of 7

Response: Thank you for your valuable suggestion. I have revised the relevant information in Supplementary **Table S3** accordingly. Please let me know if any further adjustments are needed.

2. Lines 65-67 seem to be incorrect, can you double check on this fact and explain it more comprehensively if the fact is correct (33000 maternal infections per year resulting in 57000 adverse pregnancy outcomes)?

Response: Thank you very much for your careful review and for raising this crucial point regarding the data citation in my manuscript. I fully acknowledge that my initial phrasing was imprecise and potentially misleading, and I sincerely apologize for that oversight.

Upon re-checking the primary source (Seale et al., 2017, Clinical Infectious Diseases), I confirm that the numerical data themselves are correct and that I did not misquote the numbers. However, my original sentence incorrectly implied a direct one-to-one causal progression (i.e., "33,000 maternal infections resulting in 57,000 fetal outcomes"), which is biologically and statistically inaccurate.

To clarify comprehensively for the record:

First, the 33,000 cases specifically refer to invasive GBS disease in pregnant or postpartum women (e.g., bacteremia or meningitis), which represents the most severe end of the maternal disease spectrum. As noted in the study, adult women rarely progress from vaginal colonization to systemic invasive disease (pooled

incidence: ~0.23 per 1,000 maternities, derived mainly from developed-country data). This is a conservative estimate.

Second, the 57,000 fetal infections/stillbirths are not calculated from the maternal case number. Instead, they are an independent parallel estimate derived from the prevalence of GBS detected in stillbirths (ranging from ~1% in developed regions to 4% in Africa), applied to the total global stillbirth count. Pathophysiologically, ascending GBS infection invades the amniotic cavity and targets the fetus directly. Intrauterine fetal infection and stillbirth can occur when the mother has only asymptomatic colonization or mild chorioamnionitis—without the mother necessarily progressing to systemic invasive sepsis. Therefore, it is entirely plausible and biologically consistent that the number of affected fetuses/stillbirths exceeds the number of maternal invasive cases, as the fetus is far more vulnerable to intra-amniotic infection than an adult is to bacteremia.

In light of this clarification, I have revised my statement in the revised manuscript to accurately reflect that these are two parallel adverse outcomes along the same ascending infection pathway, rather than a mathematical progression. **The revised sentence now reads:**

"Global estimates indicate an 18% colonization rate of GBS among pregnant women [1]. In 2015, an estimated 33,000 cases of invasive GBS disease occurred in pregnant or postpartum women, alongside more than 57,000 cases of fetal infection or stillbirth attributed to ascending GBS infection [2]."

3. Lines 80-83 state that Chinese guidelines recommend universal screening, but the specific indications given are not consistent with a universal screening approach. Please clarify. In addition, screening cannot be done too early in the pregnancy as colonization can fluctuate during the pregnancy.

Response: Thank you for your comment. I realize that my original wording was ambiguous and could be misinterpreted. I have revised the text to clearly describe the routine universal screening first (including the gestational age), followed by the additional special indications for intrapartum antibiotic prophylaxis. **The revised**

sentence now reads:

"Chinese clinical guidelines also recommend universal GBS screening at 35–37 weeks of gestation [11]. Additionally, pregnant women with GBS bacteriuria or a previous infant with invasive GBS disease are directly considered colonized and should receive intrapartum antibiotic prophylaxis regardless of screening results [11]."

4. Line 205 indicates a mortality rate of 8.4% to be high. Surely this is an underestimate? Please review this figure and correct or explain as appropriate.

Response: Thank you for your comment. I have rechecked the cited reference (Madrid et al., 2017), which does report a pooled global case fatality risk of 8.4% (95% CI, 6.6%–10.2%) for infant GBS disease. However, I agree that my original wording **"as high as 8.4%" was imprecise and misleading**, as this figure represents a facility-based global average and is likely a substantial underestimate of the true mortality, particularly in low-resource settings. The same study reports a CFR of 18.9% in Africa and explicitly notes that the true burden is underestimated due to unobserved community deaths. I have revised the text accordingly to clarify this point.

The revised sentence now reads:

"Vertical transmission during delivery may cause neonatal sepsis, meningitis, and pneumonia—conditions associated with a pooled case fatality risk of 8.4% [29]."

Thank you for your careful review.

Reviewer #1 (Comments for the Author):

1. To enhance clarity for the reader, I recommend explicitly stating the research question and hypothesis early in the introduction.

Response: We thank the reviewer for this valuable suggestion. In response, we have revised the first paragraph of the Introduction to explicitly state the research question at an earlier position.

Specifically, after summarizing the disease burden of GBS and the importance of prenatal screening, we have added the following sentence **at the end of the first paragraph:**

"Given the significant health impact of GBS and the crucial role of prenatal screening in guiding antibiotic prophylaxis, it is essential to ensure the accuracy and reliability of GBS diagnostic tools. However, it is unclear whether the various commercially available GBS detection kits in China perform equivalently across the range of clinically prevalent serotypes."

This revision allows readers to grasp the core research question immediately after the background introduction, while the subsequent paragraphs continue to elaborate on the specific research gaps and study objectives in greater detail. We believe this change improves the logical flow and clarity of the introduction, and we hope it satisfactorily addresses the reviewer's concern.

2. Situating the study within the existing body of knowledge is crucial. I suggest incorporating a brief review of previous relevant works to provide context for the current research.
3. The manuscript would benefit from a more detailed explanation of the challenges and gaps in the current research that this study aims to address.

Response: We sincerely thank the reviewer for the valuable and constructive comments regarding the contextualization of our study within the existing literature. We fully agree that clearly articulating the research gaps and logically deriving our study rationale from them is essential for enhancing the manuscript's impact.

In response to these suggestions, **we have substantially revised the fourth and fifth paragraph of the Introduction (lines 94-114 in the original manuscript).** Specifically, we have restructured this section to explicitly delineate three key knowledge gaps—(1) the lack of systematic investigation into serotype diversity; (2) the use of single or limited strains in prior external quality assessments; and (3) the absence of evidence on whether Chinese-marketed kits perform consistently for clinically relevant serotypes (e.g., serotype III). We then clearly articulate how our study directly addresses these gaps. This revision strengthens the logical flow from the identified shortcomings of previous works to the necessity and design of our current investigation.

The revised text is as follows:

"Most published evaluations of GBS detection methods have focused on two main aspects: comparing the sensitivity and specificity of different methodologies (e.g. culture, PCR and antigen testing) [22], and comparing the performance of various commercial diagnostic platforms using clinical specimens [23-25]. However, none of these studies have systematically investigated the impact of serotype diversity on detection performance. Secondly, while external quality assessments (EQA) have been conducted on certain commercial GBS kits [26], these evaluations have generally employed a limited number of strains and have not covered all the kits available on the Chinese market. Thirdly and most critically, no study has examined whether the detection efficiency of kits marketed in China varies for serotypes that are highly clinically relevant in China, such as serotype III. These research gaps raise concerns that some widely used kits may harbour unrecognised serotype-related analytical biases, which could lead to missed diagnoses in high-risk populations.

Therefore, a systematic, manufacturer-independent evaluation using reference materials encompassing multiple serotypes is urgently needed to objectively assess the reliability of these kits across a broad of spectrum. In this study, we employed laboratory-developed GBS reference materials representing multiple serotypes, alongside culture-based methods (the gold standard) to evaluate the performance of

the major GBS detection kits currently available in China. ***"***

We believe this revision appropriately addresses the reviewer's concerns and strengthens the scientific narrative of our manuscript. We are grateful for the opportunity to improve our work and hope this revision meets with your approval.

4. Could you please provide more specific details regarding the methodology used?

Response: In response to the request for more specific methodological details, we have revised the Methods section accordingly.

Specifically, we have **supplemented the BPL inactivation procedure** with the following information: inactivation conditions (1:2000 v/v β-propiolactone, overnight at 4 °C, followed by termination at 37 °C), and the validation of complete inactivation by plating the treated suspensions onto Columbia blood agar plates and incubating them aerobically at 37 °C for 48 hours, with no colony growth considered the criterion for successful inactivation.

We have also **clarified the result interpretation criteria**: for nucleic acid detection kits, results were interpreted based on the cycle threshold (Ct) values or amplification curve thresholds provided in each manufacturer's instructions; for immunochromatographic kits, results were read visually, and the appearance of a test (T) line was recorded as positive.

All modifications have been highlighted in the revised manuscript for easy identification. We trust that these additions adequately address the reviewers' concerns and further enhance the transparency and reproducibility of our work.

5. I noticed a few minor grammatical errors throughout the manuscript. A thorough proofread would help to improve the overall readability.

Response: We would like to thank the reviewer for their thorough and meticulous identification of the grammatical issues in our manuscript. We have taken their comments very seriously, conducting a line-by-line proofread of the entire text. We have also restructured several lengthy sentences to improve logical clarity and

readability. All revisions have been highlighted in the revised manuscript. We are confident that the revised manuscript now meets the language standards required for academic publication. Once again, we are grateful to the reviewer for their valuable suggestions.

Re: Spectrum00137-26R2 (Performance evaluation of clinical diagnostic kits for Group B streptococcus available in China)

Dear Mx. Zi-Min Tang:

Your manuscript has been accepted, and I am forwarding it to the ASM production staff for publication. Your paper will first be checked to make sure all elements meet the technical requirements. ASM staff will contact you if anything needs to be revised before copyediting and production can begin. Otherwise, you will be notified when your proofs are ready to be viewed.

Data Availability: ASM policy requires that data be available to the public upon online posting of the article, so please verify all links to sequence records, if present, and make sure that each number retrieves the full record of the data. If a new accession number is not linked or a link is broken, provide production staff with the correct URL for the record. If the accession numbers for new data are not publicly accessible before the expected online posting of the article, publication may be delayed; please contact ASM production staff immediately with the expected release date.

Publication Fees: For information on publication fees and which article types have charges, please visit our website. We have partnered with Copyright Clearance Center (CCC) to collect author charges. If fees apply to your paper, you will receive a message from no-reply@copyright.com with further instructions. For questions related to paying charges through RightsLink, please contact CCC at ASM_Support@copyright.com or toll free at +1-877-622-5543. CCC makes every attempt to respond to all emails within 24 hours.

ASM Membership: Corresponding authors may join or renew ASM membership to obtain discounts on publication fees. Need to upgrade your membership level? Please contact Customer Service at Service@asmusa.org.

Embargo Policy: A press release may be issued as soon as the manuscript is posted on the Spectrum Latest Articles webpage. The corresponding author will receive an email with the subject line "ASM Journals Author Services Notification" when the article is available online.

The ASM Journals program strives for constant improvement in our submission and publication process. Please tell us how we can improve your experience by taking this quick Author Survey.

Thank you for submitting your paper to Spectrum.

Sincerely,
Kessendri Reddy
Editor
Microbiology Spectrum
