# Supplementary material for: Performance evaluation of clinical diagnostic kits for Group B Streptococcus available in China
Source: Microbiol Spectr. 2026 Aug 27;14(10):e00137-26. doi: 10.1128/spectrum.00137-26 (PMC13689486; doi:10.1128/spectrum.00137-26)
Supplement: Supplementary file 2 [file spectrum.00137-26-s0001.docx]

**Supplementary materials**

| Table S1 | | | | | |
| --- | --- | --- | --- | --- | --- |
| Panel composition | | | | | |
| GBS | **Sample** | **Serotype** | **Source** | **Mean Ct1：adhP（3×10^8^CFU/mL）** | **One-way ANOVA** |
|  | 12386 | Ⅰa | clinic | 16.59 | P=0.3081 |
|  | g7 | Ⅰb | clinic | 16.51 |  |
|  | 27591 | Ⅰc | ATCC | 16.554 |  |
|  | g2 | Ⅱ | clinic | 16.492 |  |
|  | g3 | Ⅲ | clinic | 16.614 |  |
|  | 49446 | Ⅳ | ATCC | 16.428 |  |
|  | g5 | Ⅴ | clinic | 16.51 |  |
|  | g43 | Ⅵ | clinic | 16.62 |  |
|  | 2670 | Ⅶ | ATCC | 16.68 |  |
|  | 2669 | Ⅷ | ATCC | 16.658 |  |
|  | 2668 | Ⅸ | ATCC | 16.562 |  |
| E. coli | 25922 | / | ATCC | NC | / |

| **Table S2** | | | | | | | |
| --- | --- | --- | --- | --- | --- | --- | --- |
| **Information About the Test Kit** | | | | | | | |
| **Kit** | **Method** | **Company** | **Lysis/Extraction Method** | **Main Components of Lysis/Extraction Solution** | **Lysis/Extraction Temperature and Time** | **Detection Time** | **Sensitivity/Detection Limit** |
| **1** | Immune colloidal gold technique | HuaAo | Chemical Lysis | 2M NaNO2、  0.4M CH3COOH | Room temperature, 5-7 min | 10-20 min | ≤1×10^5^CFU/mL |
| **2** |  | Genfocus | Chemical Lysis | 2M NaNO2、  0.15M CH3COOH | Room temperature, 10 min | 10-20 min | Undetermined |
| **3** |  | Weimi | Chemical Lysis | 2M NaNO2、  1M CH3COOH | Room temperature, 5 min | 10-15 min | ≤1×10^5^CFU/mL |
| **4** | Real-time polymerase chain reaction | TIB | Heat Lysis | Tris-HCl、EDTA、Extraction solid | Dry bath at 95°C for 2 min, ice bath for 2-5 min | Stage 1: 37℃—2min Stage 2: 94℃—2min Stage 3: 94℃—20sec; 55℃—45sec; 10 cycles Stage 4: 94℃—20sec; 55℃—45sec; 30 cycles | 1×10³ copies/mL |
| **5** |  | BioPerfectus | Heat Lysis | Glass beads、Tris-HCl、EDTA | Dry bath at 99°C for 2 min, ice bath for 3 min | Stage 1: 37℃—5min Stage 2: 95℃—5min Stage 3: 95℃—10sec; 55℃—40sec; 40 cycles | 1×10^4^CFU/mL |
| **6** |  | Biotron | Magnetic Bead Extraction | / | / | Stage 1: 50℃—2min Stage 2: 95℃—20sec Stage 3: 95℃—5sec; 60℃—34sec; 45 cycles | 1000 CFU/mL |
| **7** |  | TianLong | Heat Lysis | Tris、EDTA、 NaCl、NaOH、 NP-40、Triton-100、Chelex-100 etc. | 100℃ 10min | Stage 1: 95℃—3min Stage 2: 94℃—15sec; 60℃—30sec; 40 cycles | 500 copies/mL |
| **8** |  | COYOTE | Alkaline Cleavage (no heating required) | Alkaline Solution | / | Stage 1: 95℃—5sec; 50℃—5sec; 15 cycles Stage 2: 95℃—1min Stage 3: 95℃—5sec; 58℃—30sec; 40 cycles | 500 copies/mL |
| **9** |  | DaAn Gene | Magnetic Bead Extraction | / | / | Stage 1: 50℃—2min Stage 2: 95℃—15min Stage 3: 94℃—15sec; 55℃—45sec; 40 cycles | 272 copies/mL |
| **10** |  | BioChain | Heat Lysis | Tris-HCl、NaOH、EDTA、Triton | 100℃ 10min | Stage 1: 50℃—2min Stage 2: 95℃—5min Stage 3: 95℃—15sec; 60℃—35sec; 45 cycles | 1×10^2^CFU/mL |
| **11** |  | Hybribio | Heat Lysis | Tris-HCl、KCl、phosphate buffer saline | 100℃ 10min | Stage 1: 95℃—9min Stage 2: 95℃—10sec; 58℃—50sec; 45 cycles Stage 3: 38℃—5sec | 1×10^4^CFU/mL  （or 1×10^4^ copies/mL） |
| **12** |  | Liferiver | Heat Lysis | Undetermined | 99℃ 10min | Stage 1: 37℃—2min Stage 2: 94℃—2min Stage 3: 93℃—15sec; 60℃—60sec; 40 cycles | 1×10³ copies/mL |

| Table S3 | | | | | | |
| --- | --- | --- | --- | --- | --- | --- |
| Antigen Kits Test Results | | | | | | |
| Company | **HuaAo** | | **Genfocus** | | **Weimi** | |
| Serotype | **Lower Limit Of Detection(CFU/mL)** | **Repeatability** | **Lower Limit Of Detection(CFU/mL)** | **Repeatability** | **Lower Limit Of Detection(CFU/mL)** | **Repeatability** |
| Ⅰa | 10^5^ | 20/20 | 2.10×10^7^ | 20/20 | 2.10×10^6^ | 19/20 |
| Ⅰb | 10^5^ | 20/20 | 10^7^ | 20/20 | 2.10×10^6^ | 20/20 |
| Ⅰc | 3.16×10^5^ | 20/20 | 3.16×10^7^ | 20/20 | 10^7^ | 20/20 |
| Ⅱ | 4.40×10^4^ | 20/20 | 3.16×10^6^ | 20/20 | 4.40×10^6^ | 20/20 |
| Ⅲ | 6.60×10^4^ | 20/20 | 6.60×10^7^ | 20/20 | 10^7^ | 20/20 |
| Ⅳ | 1.40×10^4^ | 20/20 | 4.40×10^6^ | 20/20 | 2.10×10^6^ | 20/20 |
| Ⅴ | 4.40×10^4^ | 20/20 | 10^7^ | 20/20 | 4.40×10^6^ | 19/20 |
| Ⅵ | 2.10×10^5^ | 20/20 | 10^7^ | 20/20 | 2.10×10^6^ | 19/20 |
| Ⅶ | 3.16×10^4^ | 20/20 | 4.40×10^6^ | 19/20 | 2.10×10^6^ | 20/20 |
| Ⅷ | 6.60×10^4^ | 20/20 | 10^7^ | 20/20 | 10^7^ | 20/20 |
| Ⅸ | 1.40×10^5^ | 20/20 | 3.16×10^6^ | 20/20 | 10^6^ | 20/20 |
| ^a^M（CFU/mL，log10） | 4.82 | | 7 | | 6.32 | |
| ^b^Q_3_ （CFU/mL，log10） | 5.15 | | 7.32 | | 7 | |
| ^c^Q_1_ （CFU/mL，log10） | 4.64 | | 6.64 | | 6.32 | |
| ^d^IQR （CFU/mL，log10） | 0.5 | | 0.68 | | 0.68 | |

**^a^M：Median；**

**^b^Q3：Quartile3；**

**^c^Q1：Quartile1**

**^d^IQR：InterQuartile Range；**

| Table S4 | | | | | | | | | | | | | | | | | |
| --- | --- | --- | --- | --- | --- | --- | --- | --- | --- | --- | --- | --- | --- | --- | --- | --- | --- |
| Q-PCR Kits Test Results | | | | | | | | | | | | | | | | | |
| Serotype | | **Lower Limit Of Detection(CFU/mL)** | | | | | | | | | | | **Comparison Of Detection Limits Of 9 Kits** | | | | |
|  | | **Ⅰa** | **Ⅰb** | **Ⅰc** | **Ⅱ** | **Ⅲ** | **Ⅳ** | **Ⅴ** | **Ⅵ** | **Ⅶ** | **Ⅷ** | **Ⅸ** | **M（CFU/mL，log10）** | **Q_3_ （CFU/mL，log10）** | **Q_1_ （CFU/mL，log10）** | **IQR （CFU/mL，log10）** | **Serotype With A Sensitivity Lower Than 10^4^ CFU/mL** |
| Company | **TIB** | 10000 | 3162 | 1000 | 316 | 3162 | 1000 | 100 | 100 | 316 | 3162 | 316 | 3 | 3.5 | 2.5 | 1 | / |
|  | **BioPerfectus** | 3162 | 1000 | 316 | 316 | 316 | 1000 | 316 | 3162 | 100 | 3162 | 316 | 2.5 | 3.5 | 2.5 | 1 | / |
|  | **Biotron** | 10000 | 10000 | 3162 | 1000 | 100 | 1000 | 316 | 1000 | 316 | 1000 | 3162 | 3 | 3.5 | 2.5 | 1 | / |
|  | **TianLong** | 316 | 1000 | 316 | 1000 | 100 | 100 | 100 | 100 | 1000 | 316 | 31 | 2.5 | 3 | 2 | 1 | / |
|  | **COYOTE** | 316 | 100 | 31 | 10 | 31 | 100 | 100 | 316 | 31 | 316 | 100 | 2 | 2.5 | 1.49 | 1.01 | / |
|  | **DaAn Gene** | 3162 | 1000 | 316 | 316 | 1000 | 1000 | 3162 | 316 | 1000 | 10000 | 1000 | 3 | 3.5 | 2.5 | 1 | / |
|  | **BioChain** | 31622 | 316 | 1000 | 3162 | 3162 | 3162 | 1000 | 100000 | 3162 | 10000 | 1000 | 3.5 | 4 | 3 | 1 | Ⅰa、Ⅵ |
|  | **Hybribio** | 31622 | 316 | 1000 | 1000 | 31622 | 100 | 1000 | 1000 | 3162 | 100000 | 1000 | 3 | 4.5 | 3 | 1.5 | Ⅰa、Ⅲ、Ⅷ |
|  | **Liferiver** | 1000 | 316 | 100 | 100 | 100 | 31622 | 3162 | 316 | 316 | 10000 | 3162 | 2.5 | 3.5 | 2 | 1.5 | Ⅳ |
| Comparison of detection limits of 11 serotypes | **M（CFU/mL，log10）** | 3.5 | 3 | 2.5 | 2.5 | 2.5 | 3 | 2.5 | 2.5 | 2.5 | 3.5 | 3 |  | | | | |
|  | **Q_3_ （CFU/mL，log10）** | 4.25 | 3.25 | 3 | 3 | 3.5 | 3.25 | 3.25 | 3.25 | 3.25 | 4 | 3.5 |  |  |  |  |  |
|  | **Q_1_ （CFU/mL，log10）** | 2.75 | 2.5 | 2.25 | 2.25 | 2 | 2 | 2 | 2.25 | 2.25 | 2.75 | 2.25 |  |  |  |  |  |
|  | **IQR （CFU/mL，log10）** | 1.5 | 0.75 | 0.75 | 0.75 | 1.5 | 1.25 | 1.25 | 1 | 1 | 1.25 | 1.25 |  |  |  |  |  |
|  | **Test Kits With A Sensitivity Lower Than 10^4^ CFU/mL** | BioChain、Hybribio |  |  |  | Hybribio | Liferiver |  | BioChain |  | Hybribio |  |  |  |  |  |  |

**Figure S1.** Comparable CT values（n=5） of housekeeping gene adhP (mean = 16.57) across 11 GBS serotypes (P = 0.3081).
